# Clinical features and familial mutations in the coexistence of Wilson's disease and Alport syndrome: A case report

**DOI:** 10.3389/fped.2023.1107280

**Published:** 2023-03-31

**Authors:** Ying Wang, Qingnan He, Xiqiang Dang, Xiaochuan Wu, Xiaoyan Li

**Affiliations:** ^1^ Department of Pediatrics, The Second Xiangya Hospital, Central South University, Changsha, China; ^2^ Department of Pediatrics Nephrology, Children’s Medical Center, The Second Xiangya Hospital, Central South University, Changsha, China; ^3^ Department of Pediatrics, The Third Xiangya Hospital, Central South University, Changsha, China

**Keywords:** Alport syndrome, *COL4A5*, Wilson's disease, *ATP7B*, proteinuria, hematuria

## Abstract

**Background:**

Alport syndrome (AS) and Wilson's disease (WD) are genetic diseases that could lead to kidney damage. Herein, we report the clinical features and gene variants in a patient with WD and X-linked AS.

**Case presentation:**

The proband was a 12-year-old boy diagnosed with AS coexisting with WD at the age of 11 years. The patient underwent a medical check-up when he was 4 years and 8 months. Laboratory tests revealed elevated liver enzymes, decreased serum ceruloplasmin, increased 24-h urinary copper excretion, and one variant in the *ATP7B* gene. Then, the patient was diagnosed with WD. After 2 months of treatment with D-penicillamine and zinc salt, his liver function had recovered to normal levels, but he presented with microscopic hematuria. The hematuria did not resolve after switching to dimercaptosuccinic acid from D-penicillamine. In addition, he presented with proteinuria 3 years later. A renal biopsy was performed more than 6 years after the patient was diagnosed with WD, and electron microscopy showed that the basement membrane thickness was uneven, layered, and focal torn. Copper staining was negative. A genetic analysis identified a hemizygous variant (c.1718G > A, p. Gly573Asp) in *COL4A5* and a homozygous variant (c.2975C > T, p. Pro992leu) in *ATP7B*. The patient’s urine protein–creatinine ratio was less than 1.0 mg/mg after a 1 year of follow-up, after enalapril was administered for treating AS.

**Conclusion:**

This case highlights a lack of improvement in renal function after conventional treatment provides a possible indication for performing renal biopsy or genetic testing to determine the etiology in order to facilitate subsequent clinical management. Clinicians should prevent the occurrence of diagnostic inaccuracies caused by diagnostic anchoring because an accurate diagnosis is essential for achieving precise treatment and improved prognosis.

## Introduction

Alport syndrome (AS) and Wilson's disease (WD) are both causes of painless hematuria and proteinuria. AS is among the most prevalent inherited kidney diseases and is even more frequent than autosomal dominant polycystic kidney disease (1, 2). AS is characterized by hematuria, proteinuria, and progressive renal failure. In addition, AS is associated with extrarenal manifestations such as ocular abnormalities and sensorineural deafness (1–5). It is related to *COL4A3*, *COL4A4*, and *COL4A5* gene mutations, which encode for the α3, α4, and α5 chains of type IV collagen, respectively. Type IV collagen is the principal constituent of basement membranes of the glomerular basement membrane (GBM), retina, cornea, lens capsule, and cochlea (1, 6). WD, also referred to as hepatolenticular degeneration, is a sporadic autosomal recessive hereditary disorder disease. *ATP7B* gene mutation reduces the function of copper-transporting *P*-type ATPase, leading to impaired serum ceruloplasmin synthesis, biliary copper excretion, and systemic copper overload and deposition in multiple organs, including the liver, brain, cornea, kidney, bone, and joint (7–9). Hepatic, neurologic, and psychiatric disorders and Kayser–Fleischer (K–F) rings at the corneal limbus are the most common clinical manifestations of WD. Hemolytic anemia, renal damage, and rheumatological manifestations can also occur in WD, but renal involvement is relatively rare (8, 9). The reported possible causes of abnormal urine analysis in WD patients include the deposition of copper and/or immune complex, adverse drug-related events, hypercalcinuria, and abnormal blood coagulation secondary to hepatic dysfunction. (7, 10, 11). Instances of AS and WD occurring simultaneously or successively in the same patient are rare (12). Herein, we report the case of a Chinese boy who presented with liver function damage, hematuria, and proteinuria successively. His kidney biopsy showed the characteristic GBM lesion of Alport 6 years after being diagnosed with WD and a hemizygous c.1718G > A (p. Gly573Asp) variant in *COL4A5* and a homozygous c.2975C > T (p. Pro992leu) variant in the *ATP7B* gene were found.

## Case presentation

**Figure 1:**
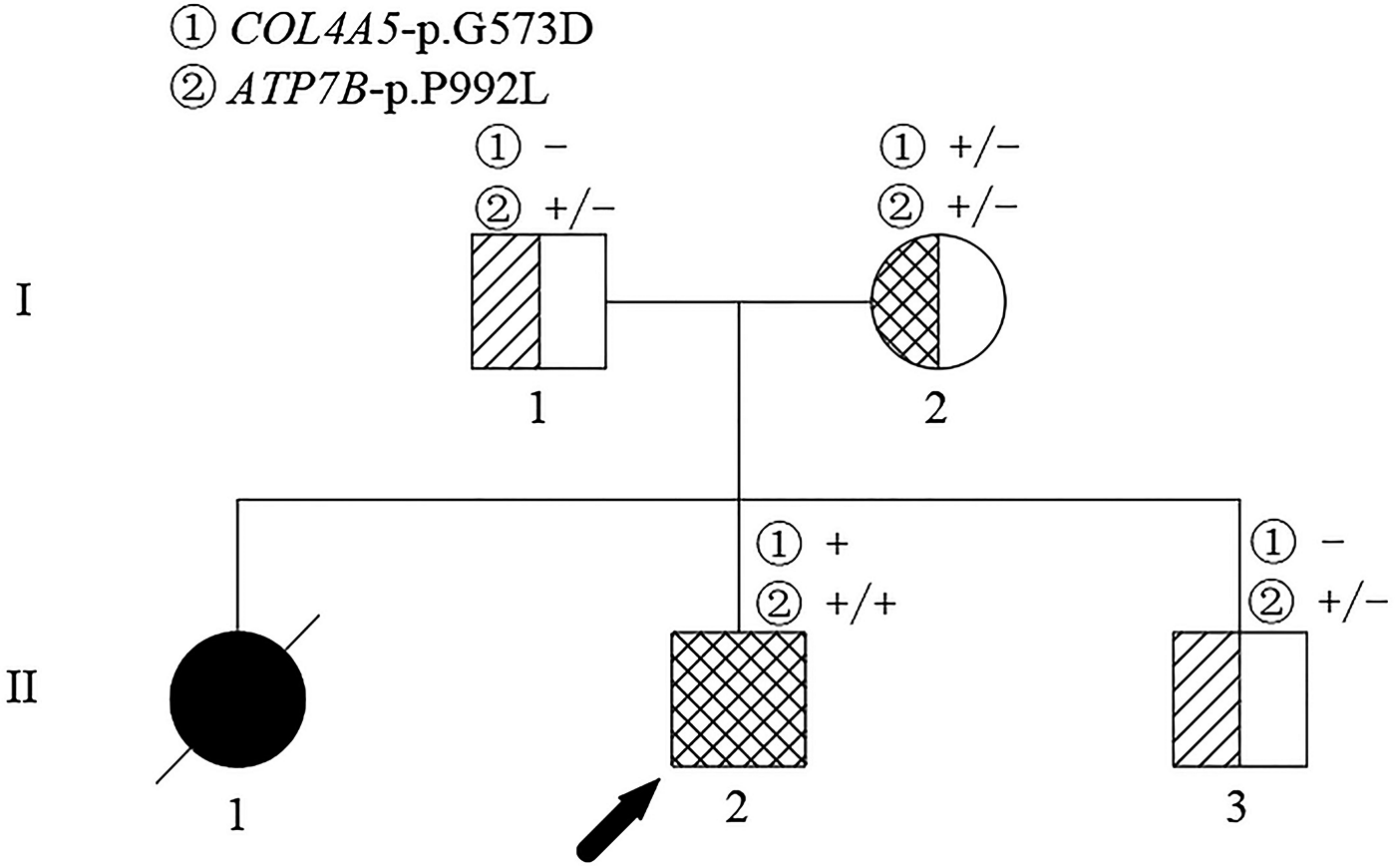
Pedigree of the family affected. The arrow identifies the proband. The crossed symbol indicates the deceased individual.

The proband (II-2 in Figure 1) was a 12-year-old boy diagnosed with AS coexisting with WD at the age of 11 years. The patient was the second child of a non-consanguineous couple of Chinese Han ethnicity. The visual and auditory functions, hepatic and renal functions, and urinalysis of the patient's parents (I-1, I-2 in Figure 1) and younger brother (II-3 in Figure 1) were normal. However, the patient's elder sister experienced severe ascites and liver dysfunction with a clinical suspicion of WD and died at the age of 7 years. The patient underwent a medical checkup following the death of his elder sister when he was 4 years and 8 months. Laboratory tests revealed elevated liver enzymes (alanine aminotransferase >100 IU/L), decreased serum ceruloplasmin (122 mg/L), and elevated 24-h urinary copper excretion (93.3 µg/24 h), but the K–F ring was not observed on a slit lamp examination. In addition, a variant in the *ATP7B* gene was found, but specific information related to the mutations was unavailable. The patient was diagnosed with WD and treated with D-penicillamine (DPA) and vitamin B6 combined with zinc salt at local hospitals. At 4 years and 10 months of age, his liver function recovered to normal levels, but he presented with microscopic hematuria. Because his doctor believed that hematuria was caused by DPA, DPA was replaced with dimercaptosuccinic acid (DMSA). However, the patient's hematuria persisted and he presented with severe proteinuria at the age of 8 years, despite discontinuing DPA. The symptoms of hematuria and proteinuria persisted and there was no improvement in his condition. Liver enzyme levels were maintained about twice the upper limit of normal under DMSA and zinc salt treatment in the following years.

**Figure 2:**
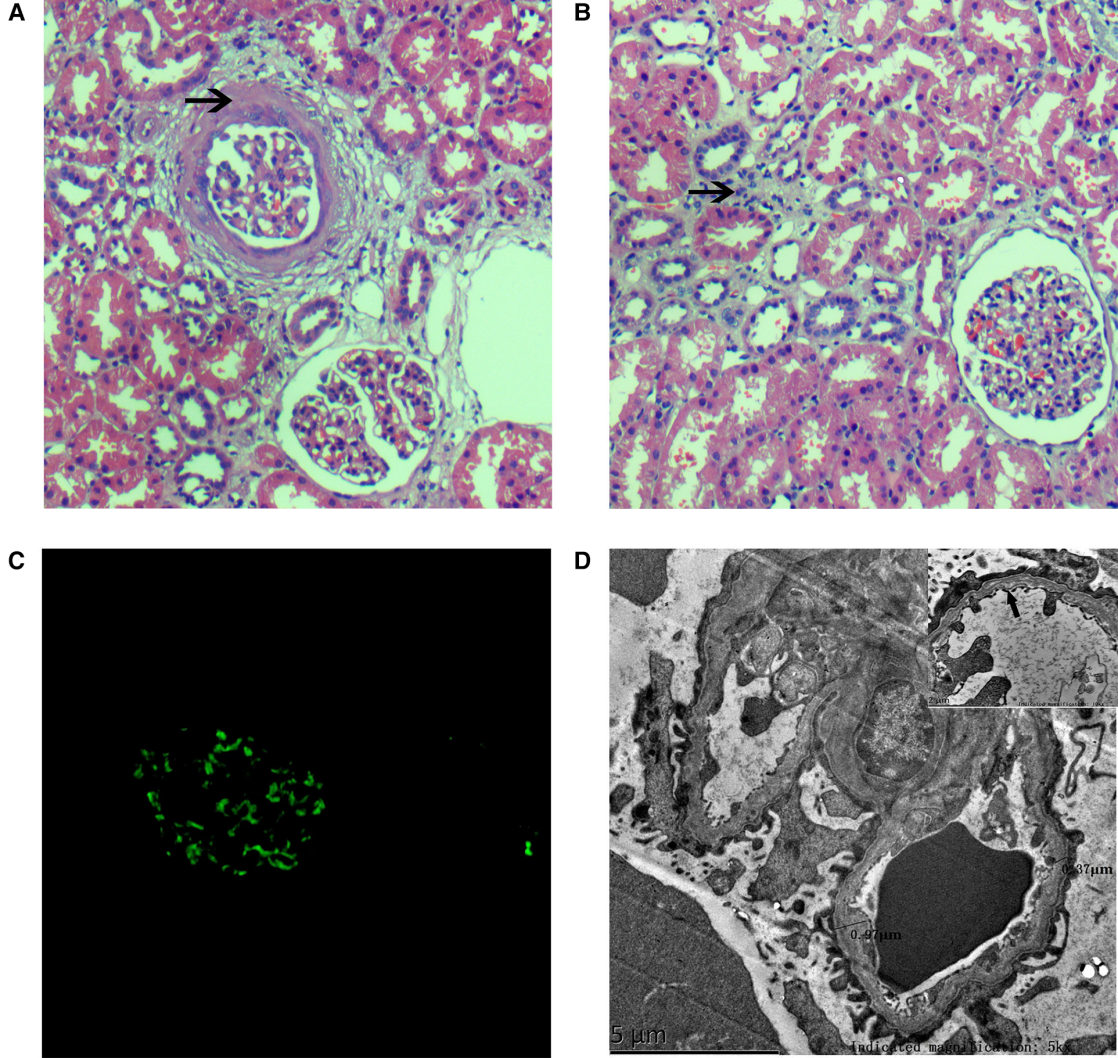
Histopathologic lesions. (**A**) H&E stain (×200) showing glomeruli mesangial hypercellularity, a thickened mesangial matrix, and a ring-like mixed crescent form (↑); (**B**) the renal tubules depicting interstitial inflammatory cell infiltration and interstitial fibrosis (↑) by a light microscopy study (H&E stain, ×200); (**C**) immunofluorescence staining showing granular deposition of IgM (++) within the mesangium; and (**D**) electron micrograph showing abnormal glomerular capillary basement membrane thickness, uneven thickness, (ranging from 350 to 900 nm), layered (↑), focal torn, and endothelial cell insertion.

At the age of 11 years, the patient was admitted to our hospital for further evaluation. On admission, his physical examination showed a body temperature of 36.3°C, pulse rate of 120 /min, respiration rate of 28 cycles/min, blood pressure at 85/50 mmHg, and weight of 38 kg. Percussion pain was noted in the left kidney area, with no other abnormalities on examination of the abdomen. The patient did not have edema or a yellow-tinged skin, and the results of cardiac, pulmonary, and neurological examinations were normal. Laboratory tests after admission showed an abnormal liver function and blood lipid level: albumin (29.5 g/L, normal range: 40.0–55.0 g/L), alanine aminotransferase (111.6 U/L, normal range: 9–50 U/L), aspartate aminotransferase (47.3 U/L, normal range: 15–40 U/L), cholesterol level (6.41 mmol/L, normal range: 2.9–5.2 mmol/L), and triglyceride levels (4.25 mmol/L, normal range: 0.0–1.71 mmol/L). Further workup due to chronic abnormal liver enzymes showed a serum ceruloplasmin level of 40 mg/L, and 24-h urinary copper excretion was 251 µg/24 h. In addition, routine urine tests showed proteinuria (3+) and red blood cells (2+), and 24-h uric protein quantity was 3,823.3 mg/day. Renal function was normal. Other laboratory examinations showed a slight decline in IgG (6.06 g/L, normal range: 8.60–17.40 g/L) and a mildly elevated complement C3 (1.61 g/L, normal range: 0.70–1.40). His serum electrolytes, C-reactive protein, serum complement C4, antistreptolysin O, immunoglobulin levels (IgA, IgE, and IgM), coagulation function, antinuclear antibody, anti-double-stranded DNA antibody, antineutrophil cytoplasmic antibody, anti-GBM antibody, interferon-gamma release assay, and serologic hepatitis B virus and hepatitis C virus testing were all normal. No obvious abnormalities were noted upon ophthalmologic and hearing screening. In addition, the results of routine chest radiograph, electrocardiogram, color echocardiogram, double kidney color ultrasound, and cranial magnetic resonance imaging were normal. Color ultrasound of the liver, gallbladder, spleen, and pancreas indicated intrahepatic fat deposition and gallbladder stones. Renal biopsy was performed to identify whether there was kidney disease. An analysis of renal biopsy specimens using light microscopy showed the formation of annular cellular and fibrous crescents in three glomeruli (3/16 glomerulus) (Figure 2A), hypertrophy of other glomeruli, increased number of intrinsic cells, lobulation of glomeruli with mesangial hypercellularity, segmental thickening of the mesangial matrix and a small amount of redophilic deposits in the mesangial area, vacuolated renal tubular epithelial cells and the presence of casts in the tubular lumen, interstitial focal inflammatory cell infiltration, interstitial fibrosis, and mild edema (Figure 2B). Immunofluorescence staining of kidney tissue revealed granular deposition of IgM (++) within the mesangium (Figure 2C) and showed negativity for IgG, IgA, C3, C4, C1q, and Fibri. Detection of the α5 collagen chain of the basement membrane was not performed. Copper staining was negative. Electron microscopy showed a mild to moderate proliferation of the mesangial matrix, occasionally seen as electron-dense deposits in the glomerular mesangial area. Electron microscopy also revealed an abnormal glomerular capillary basement membrane thickness, which was uneven (ranging from 350 to 900 nm) or layered, focal torn, and endothelial cell insertion; and multiple lysosomes in the podocyte cytoplasm, with widely fused foot processes (Figure 2D). The prominent renal pathological changes observed suggested that a diagnosis of AS needs to be considered.

**Figure 3:**
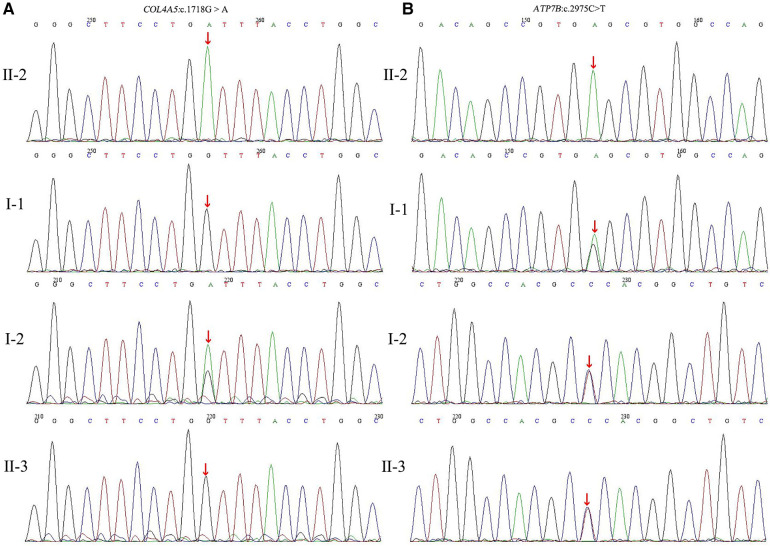
(**A**) Partial DNA sequences in the *COL4A5* gene of the family. The proband (II-2) inherited hemizygous variation (c.1718G > A). The proband's mother (I-2) carried the c.1718G > A heterozygous variant, but this gene was normal in the proband's father (I-1) and younger brother (II-3); (**B**) Partial DNA sequences in the *ATP7B* gene of the family. The proband carried the homozygous mutation (c.2975C > T). The proband's father, mother, and younger brother carried the c.2975C > T heterozygous variants.

At this point, peripheral blood samples collected from the proband were sent to the MyGenostics Medical Laboratory (Beijing, China) for whole-exome sequencing and for further confirmation of the results by Sanger sequencing. A hemizygous c.1718G > A (NM_000495) coding variant (p. Gly573Asp) in exon 24 of the *COL4A5* gene located on the X-chromosome and a homozygous variant c.2975C > T (p. Pro992Leu) in exon 13 of the *ATP7B* (NM_000053) gene located on chromosome 13 were identified. Familial verification was performed by using Sanger sequencing. The results showed that the same heterozygous *COL4A5* variant was detected in his mother (Figure 3A). The same heterozygous missense mutation of *ATP7B* was detected in his parents and younger brother (Figure 3B). This p. Gly573Asp variant in the *COL4A5* gene has been reported previously (13). It is listed in the Human Gene Mutation Database (HGMD) and is believed to cause AS (accession number CM983307). The p. Pro992Leu variant in *ATP7B* is located in the hot spot region (14) and is one of the common *ATP7B* mutations in Chinese patients with WD (autosomal recessive) (15). These two variants showed an extremely low frequency in the general population cohort, and the Rare Exome Variant Ensemble Learner (REVEL) was used to predict possible damage to protein function. Thus, the c.1718G > A (NM_000495) and c.2975C > T (NM_000053) variants were classified as “likely pathogenic (PS4 + PM2 + PP3)” and “pathogenic (PM1 + PM2 + PM3_Strong + PM5 + PP3),” respectively, according to ACMG/AMP guidelines (16) and the Sequence Variant Interpretation Working Group general recommendations for using ACMG/AMP criteria (https://clinicalgenome.org/working-groups/sequence-variant-interpretation/).

**Figure 4:**
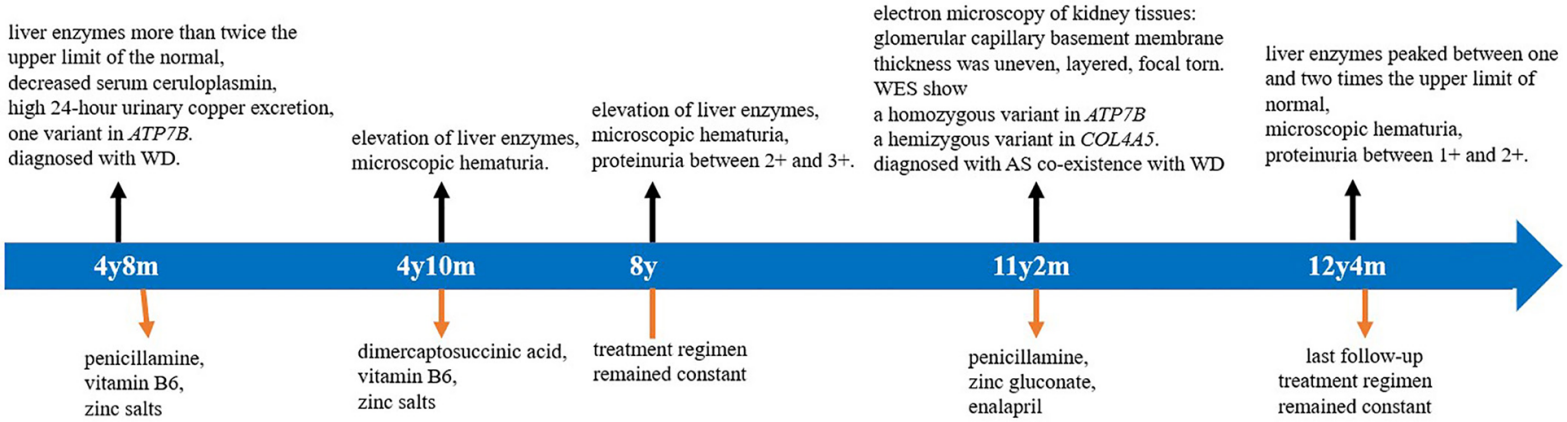
Timeline of clinical course, diagnostics, and treatment regimen of the patient. WES, whole-exome sequencing; AS, Alport syndrome; and WD, Wilson's disease.

Based on the clinical manifestations, renal pathological characteristics, and gene sequencing results, the patient was diagnosed with AS and WD. The patient was treated with DPA (125 mg orally twice a day), zinc gluconate (210 mg orally twice a day), and enalapril (10 mg orally once a day). Follow-up urinalysis showed proteinuria between 1 + and 2 +, urinary erythrocyte between 1 + and 2 + , and urine protein–creatinine ratio between 0.2 and 1.0 mg/mg during the 1-year follow-up period after genetic diagnosis. Biochemical analyses showed that liver enzymes were between one and two times the upper normal limit, and renal function was normal. The treatment was well tolerated, with good compliance. No adverse events were observed. The patient was able to maintain a good quality of life. A timeline of the clinical course, diagnostics, and treatment regimen of the patient are summarized in Figure 4.

## Discussion

Although AS can be diagnosed with high accuracy because of an improved understanding of the disease and the availability of molecular diagnostic testing, misdiagnosis or missed diagnosis of AS is not rare, especially when it coexists with other diseases with renal manifestations, such as WD. In this report, we report a Chinese boy with WD presenting with microscopic hematuria and proteinuria. He received a kidney biopsy, which showed the characteristic GBM lesion of Alport 6 years after being diagnosed with WD. Whole-exome sequencing was performed and a hemizygous variant c.1718G > A (p. Gly573Asp) in *COL4A5* and a homozygous variant c.2975C > T (p. Pro992leu) in *ATP7B* were found. Therefore, reports of such unique cases may improve awareness of the disease.

WD is an autosomal recessive disease characterized by the *ATP7B* gene mutation, resulting in an abnormal biliary excretion of copper and excessive copper accumulation in multiple tissues, thus causing cellular damage and corresponding clinical symptoms of the involved system. Clinical signs and symptoms of the disease are diverse, including hepatic disorders, neurologic and psychiatric disorders, renal manifestation, ophthalmic signs (Kayser-Fleischer rings), bone-muscular alterations, episodes of hemolysis, cardiovascular involvement, altered skin pigmentation and a bluish color at the base of the nails (17). To date, more than 700 mutations have been identified in the *ATP7B* gene, *ATP7B* hotspot mutations vary greatly among regions, and p.Arg778Leu, p.Pro992Leu, and p.Thr935Met are common *ATP7B* mutations in Chinese patients with WD (15). *ATP7B* is also expressed in the kidneys (10). A wide variety of renal abnormalities have been described in WD (18), including nephrotic syndrome, glomerulonephritis, IgA nephropathy (9, 19, 20), IgM nephropathy (21), tubular dysfunction, and renal calculis (22). In a retrospective study, Zhuang et al. analyzed 25 patients with WD-related renal impairment who were not treated with DPA. Among these 25 patients, five had elevated urine N-acetyl-β-D-glucosaminidase, six had increased urine β2-microglobulin, and five had both proteinuria and hematuria (23). Although the mechanism of renal damage of WD is unknown, copper has mainly been recognized to deposit in the epithelium of the proximal and distal convoluted tubules, leading to a thickening of the basement membrane and interfering with the reabsorption function of the renal tubules (10). However, copper staining has not been used to diagnose renal pathology in many people. In a case report (20), a renal assessment demonstrated IgA nephropathy with no tubular damage or copper deposition. This observation may be attributed to the liver's inability to clear the immunoglobulin and immune complexes, resulting in their increased levels in the systemic circulation and accumulation in the kidneys and leading to nephropathy. The cause of non-glomerular hematuria may be hypercalcinuria or coagulopathy (23). In addition, the adverse drug reactions caused by DPA should be taken into consideration. Patients who are on treatment with DPA may develop proteinuria, hematuria, Goodpasture syndrome, severe fatal glomerulonephritis associated with intraalveolar hemorrhage, and renal vasculitis (11, 23). The deposition of copper, the adverse effects of drugs, and hepatic dysfunction are the causative factors of renal impairment in WD.

The timing of renal biopsy is very important for making a definite diagnosis, providing treatment guidance, and estimating prognosis. The clinical manifestations, abnormal copper metabolism, and homozygous mutations in the *ATP7B* gene of our proband were consistent with the diagnosis of WD. The patient presented to our nephrology department with abnormal urine test results of over 6 years since his diagnosis of WD, with such results always believed to have been attributed to WD. AS was suspected after electron microscopy revealed that the GBM in the renal tissue was of uneven thickness, layered, and focal torn. Although we did not use immunohistochemistry or immunofluorescence to assess the expression of the type IV collagen α5 chain protein in the GBM, subsequent gene analyses of the *COL4A5* mutation of the patient supported the diagnosis of AS. In our study, we comprehensively examined the history of a two-generation family. We performed whole-exome sequencing for the proband and used Sanger sequencing to verify the pedigree. The proband was found to have a hemizygous c.1718G > A coding variant (p. Gly573Asp) in exon 24 of the *COL4A5* gene located on the X-chromosome and genetic analysis identified the same X-linked mutation in his mother. However, his mother’s urine test result or kidney function was not abnormal. X-linked inheritance of *COL4A5* mutations accounts for 85% of AS cases (24). Hematuria has been reported to be highly penetrant with pathogenic variants of *COL4A5*. Hematuria occurs in almost all men and 95% of women with the variant and is consistently absent in the remaining 5% of women (25). According to the 2020 clinical practice recommendations for AS (4), girls and women with *COL4A5* variants are labeled to have AS rather than “carriers” and are at risk for progressive kidney disease. The diagnosis of AS in the proband and his mother was established according to the clinical practice recommendations (4). IgM deposits in the mesangial area were also observed in our patient, but IgM nephropathy has been described in WD (21) and AS (26, 27). Our patient's clinical laboratory data and gene mutations were consistent with AS and WD.

X-linked and autosomal recessive AS are associated with the greatest risk of kidney failure (2). About 90% of men with a pathogenic variant of the *COL4A5* gene develop kidney failure by 40 years of age (2). Early diagnosis is important because of the potentially substantial benefit associated with early diagnosis of AS. The risk of progression to kidney failure in AS patients can be modified with early intervention. Higher GFRs at treatment initiation are associated with greater delays in the progression to kidney failure (5). The most effective therapy for AS is initiating an angiotensin-converting enzyme inhibitor (ACEi) before kidney function starts to decline. It delays the need for kidney replacement therapy for several years and even decades. Clinical practice recommends initiating treatment at the time of diagnosis in males with X-linked AS and at the onset of microalbuminuria in females with X-linked AS (4). In our current case, the patient received ACEi for AS and a therapeutic regimen for WD after the final diagnosis was determined. The patient was observed with decreased urinary protein, normal renal function, and no adverse events after treatment. The patient's family members were satisfied with the clear diagnosis made but expected better treatment effects, such as negative proteinuria. We explained in detail to the patient's family that the goal of treatment is to delay the onset of kidney failure. In addition, the patient's family was informed that if his urine protein–creatinine ratio remains greater than 1.0 despite maximum ACEi dosing, an angiotensin receptor blocker or an aldosterone antagonist will be added to his treatment (4).

When WD patients present with abnormal urine test results, a repeat 24-h urine copper test is advised to check the adequacy of treatment and also consider the adverse effects secondary to DPA administration. Even after maintaining urinary copper excretion between 200 and 500 µg/24 h (15) and after discontinuing DPA treatment, the urinalysis does not shown normal results, a kidney biopsy and the possibility of other kidney diseases should be considered. Anchoring bias is a common cognitive bias in medicine and is associated with therapeutic or management inaccuracies (28). In our patient, hematuria occurred after 2 months of DPA treatment, which was believed to be a fallout of this treatment. But hematuria had not remitted and proteinuria occurred approximately 3 years later after DPA was replaced with DMSA to control WD, and the clinicians attributed the renal damage to a WD-associated one. The last physician in the group avoided diagnostic anchoring and made an accurate diagnosis.

## Conclusion

In summary, this case report highlights that prompt kidney biopsy should be performed in patients with WD and renal impairment who do not respond well to conventional treatment. In addition, genetic testing may be required to be performed on the basis of biopsy results. Clinicians have to be alert to errors caused by diagnostic anchoring.

## Data Availability

The original contributions presented in the study are included in the article/Supplementary Material; further inquiries can be directed to the corresponding author.
